# Emergence and impact of theranostic‐nanoformulation of triple therapeutics for combination cancer therapy

**DOI:** 10.1002/SMMD.20230035

**Published:** 2024-01-30

**Authors:** Amit Kumar Rajora, Eknath D. Ahire, Manju Rajora, Sukhvir Singh, Jaydeep Bhattacharya, Hongbo Zhang

**Affiliations:** ^1^ NanoBiotechnology Lab School of Biotechnology Jawaharlal Nehru University New Delhi India; ^2^ Department of Pharmaceutics, Mumbai Educational Trust (MET), Institute of Pharmacy Affiliated to Savitribai Phule, Pune University Nashik Maharashtra India; ^3^ College of Nursing All India Institute of Medical Sciences New Delhi India; ^4^ Radiological Physics and Internal Dosimetry (RAPID) Group Institute of Nuclear Medicine and Allied Sciences Defense Research & Development Organization, Ministry of Defense Timarpur Delhi India; ^5^ Pharmaceutical Sciences Laboratory Faculty of Science and Engineering Åbo Akademi University Turku Finland; ^6^ Turku Bioscience Center University of Turku and Åbo Akademi University Turku Finland

**Keywords:** cancer, cancer treatment, imaging, theranostic nanoformulation, triple therapeutics

## Abstract

Cancer remains a major global health threat necessitating the multipronged approaches for its prevention and management. Traditional approaches in the form of chemotherapy, surgery, and radiotherapy are often encountered with poor patient outcomes evidenced by high mortality and morbidity, compelling the need for precision medicine for cancer patients to enable personalized and targeted cancer treatment. There has been an emergence of smart multimodal theranostic nanoformulation for triple combination cancer therapy in the last few years, which dramatically enhances the overall safety of the nanoformulation for in vivo and potential clinical applications with minimal toxicity. However, it is imperative to gain insight into the limitations of this system in terms of clinical translation, cost‐effectiveness, accessibility, and multidisciplinary collaboration. This review paper aims to highlight and compare the impact of the recent theranostic nanoformulations of triple therapeutics in a single nanocarrier for effective management of cancer and provide a new dimension for diagnostic and treatment simultaneously.

## Graphical abstract

This review paper highlights the significance of nanotheranostic for the treatment of cancer. Nanotheranostic is a treatment strategy in which treatment is guided by imaging techniques using nanotechnology. The treatment of cancer in pre‐clinical application in mice is employed through triple therapeutics combinations (e.g, combination of chemotherapy, immunotherapy, radiotherapy and phototherapy) guided by imaging (e.g, computerized tomography, photoacoustic imaging and magnetic resonance imaging) in a single nanocarrier. These theranostic nanoformuations have demonstrated sustained drug targeting, minimal toxicity, and overall higher safety.

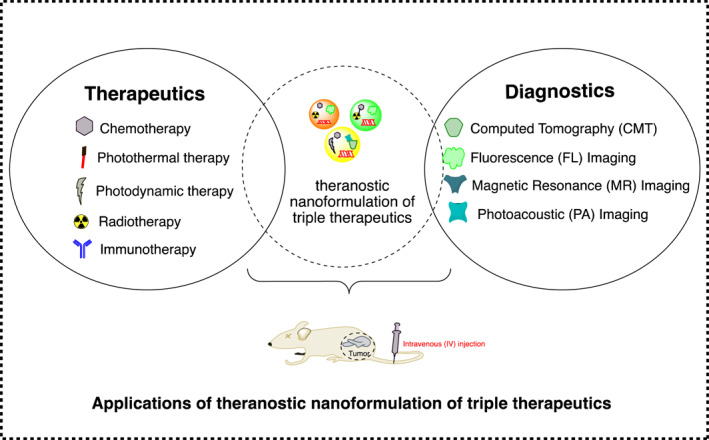

**Key points**

- Emerging theranostic nanoformulations of triple therapeutics cancer treatment are discussed.
- Impact of theranostic nanoformulations of triple therapeutics for cancer management is reviewed.
- Future outlook of current theranostic nanoformulations of triple therapeutics is highlighted.

## 1 INTRODUCTION

Globally, cancer is a serious burden and a threat to public health and is one of the leading causes of death.^1^, ^2^, ^3^ It also predominates among the healthcare challenges for improving public health.^4^, ^5^, ^6^, ^7^ Particularly in India, 14, 61, 427 cancer patients were reported in 2022 and about 50% have lost their lives due to the poor prognosis of cancer, especially at advanced staged metastasis (cancer statistics, 2022, India).^8^, ^9^, ^10^, ^11^ The number of new cases of cancer would likely increase at national and international levels due to the high prevalence and burden of modifiable risk factors, including obesity, sedentary lifestyle, poor eating habits, lack of exercise, and smoking.^12^ In the long run, cancer leads to poor patient outcomes in terms of morbidity, mortality and higher cost to patients and healthcare.^13^, ^14^, ^15^ For cancer management, an early diagnosis, targeted treatment with minimal toxicity and prevention of recurrence and metastasis are the key objectives.^16^, ^17^, ^18^, ^19^, ^20^, ^21^, ^22^, ^23^, ^24^, ^25^

There are various treatment options for cancer, including chemotherapy, radiotherapy (RT), surgery^9^ and immunotherapy (IT).^26^, ^27^, ^28^, ^29^, ^30^, ^31^, ^32^, ^33^, ^34^, ^35^, ^36^, ^37^ However, each option has its own limitations. Chemotherapy comes with multi‐drug resistance; RT damages the surrounding tissue limiting their applications and post‐surgical treatment many patients are left with positive tumor margin and poor specificity. Immunotherapy, also known as biotherapy or biological response modifier therapy, is a path breaking treatment for cancer, wherein the body uses its own immune power to kill the cancer cells. Although IT is not suitable for all types of cancer, it can be used for certain types of cancer (lymphoma and leukemia), particularly the metastasis cancer, which didn't see any hopes for last many decades. Immunotherapy can be used as a single therapy for cancer treatment, or it can be used in combination with other treatment options such as surgery, chemotherapy, or RT. Immunotherapy, is an innovative cancer treatment in today's scenario with prolonged progression‐free survival and overall survival;^38^ however ,it has certain uncertainties and may cause serious adverse reactions. Therefore, the treatment needs to be highly selective and effective with minimal side effects.^39^, ^40^, ^41^, ^42^, ^43^, ^44^

Theranostic is a term encompassing therapeutic and diagnostic imaging, coined by Funkhouser in 2002.^45^, ^46^ Theranostic includes delivering the drugs and imaging agents at the same time with a particular dosage aimed to monitor drug delivery and drug release, and achieve highest therapeutic efficacy.^47^ In pre‐clinical models, real‐time feedback on pharmacokinetics, drug targeting, and off‐targeting of healthy organs have been reported in theranostic nanoformulations.^48^ In the last few decades, multifunctional nanoformulation‐based combination therapy guided by imaging (such as computerized tomography [CMT], magnetic resonance [MR] imaging, Positron emission tomography [PET], ultrasound, fluorescence [FL], and photoacoustic [PA]), has grasped the attention of researchers to overcome multidrug resistance, minimize drug toxicity and overall enhancing the anti‐tumor efficacy, however, internal synergism remains a concern due to varying pharmacokinetics of each drug.^48^, ^49^, ^50^, ^51^, ^52^, ^53^, ^54^, ^55^ Therefore, to overcome this limitation, theranostic nanoformulation approach with the combination of triple therapeutics (any three therapeutics) into one nanoplatform has considerably taken a center stage among monotherapy of cancer such as chemotherapy, RT, IT, and phototherapy.^56^, ^57^, ^58^, ^59^, ^60^, ^61^, ^62^, ^63^, ^64^

RT, for a long time, has been one of the primary treatment options for cancer; however, if given as the only treatment, it is not effective against the hypoxic tumor cells, causing a major setback on its own as a therapy. Thus, to enhance the RT efficacy, radio‐therapeutic sensitizers need to be added during RT to improve oxygen concentration in tumor microenvironment (TME)^32^, ^33^, ^34^, ^65^ or RT to be combined with other therapeutic modalities (e.g., RT/photodynamic therapy [PDT] or RT/photothermal therapy [PTT]) to improve the treatment effectiveness. Some of the reported radio‐sensitizers based on nanoparticles (NPs) include gold (Au), tantalum oxide (TaOx), topological insulator bismuth selenide (Bi_2_Se_3_), tungsten disulphide (WS_2_), and polyoxometalates and have shown great results in RT efficacy.^66^, ^67^, ^68^, ^69^ Phototherapy administered in the form of PDT or PTT has great potential and is being employed as a therapy due to its minimally invasive effect and spatiotemporal selectivity. PDT similar to RT depends on photosensitizer (PS) for its maximum therapeutic efficacy by generating reactive oxygen species (ROS)^59^, ^70^ for example, singlet oxygen (^1^O_2_) leading to cancer cells death; however, hypoxic TME greatly narrows the scope of ^1^O_2_. Furthermore, the higher concentration of oxygen during PDT may further inhibit the anti‐tumor response. Other techniques such as high laser and prolonged PDT have been tested to overcome this issue, unfortunately it damages the normal tissue and hence limiting PDT application in clinical setting. Moreover, PDT is not effective for deep‐seated tumor due to poor light penetration into deeper tissues. Therefore, an alternative approach is warranted to increase the therapeutic efficacy. In recent years, porphyrinic metal‐organic based scaffold (PMOF) nanoparticles are established as next‐generation PDT system with great potential. PMOF containing the porphyrins are dispersed to prevent self‐quenching from PS aggregation and hence support the diffusion of photogenerated ROS and eventually enhance the PDT effect. In addition, these NPs can act as nanocarriers for encapsulating multiple therapeutic agents (antineoplastic drugs and photothermal reagents) to achieve synergism.^71^ On the other hand, PTT employs the use of heat produced from absorbed near‐infrared rays to “cook” the most hypoxic tumor cells. Further, PTT‐induced hyperthermia promotes the photosensitizer accumulation into tumor cells and increases oxygen supply in the tumor; hence, if combined with PDT and RT, it increases the effectiveness through a synergistic action, rather than PTT^57^, ^58^, ^72^ alone, particularly for deep‐seated tumor. Hence, combined photo and radiotherapy can be exploited as a very effective cancer treatment option by using each therapy advantage and remedy in overcoming the drawbacks.^67^, ^68^ There are various imaging techniques such as photoacoustic (PA), PET, computerized tomography (CMT),^73^, ^74^ MR imaging, single photon emission computed tomography (SPECT), fluorescence imaging (FL) and two‐photon excited fluorescence imaging, with different potential for providing information about the structural and functional aspect of an organ, are being currently used for biomedical and medical imaging^75^, ^76^, ^77^ (features of the imaging modalities are compared in Table 1).

**TABLE 1:** A comparison of features of distinct imaging modalities for tumor diagnosis.

Imaging techniques	Type of imaging modality (source)	Typical probes	Resolution (sensitivity)	Depth	Time	Pros	Con	Information (clinical use)	Ref
X‐rays computed tomography (CMT)	CMT (X‐rays)	Heavy metal (iodine, barium, Krypton, Xenon and tungsten used as contrast agent)	−0.5 mm (millimolar level)	No limit	Minutes	Fast and high spatial resolution	Poor soft tissue penetration, risk from contrast agent and ionising radiations, low sensitivity	3D extension of conventional X‐rays (2D), which gives clear view by cross sectional images anatomically and physiologically (yes)	78, 79, 80
Magnetic resonance imaging (MRI)	MRI (radio wave and magnetic field)	Paramagnetic and ferromagnetic atoms (gadolinium Gd, Mn), iron oxide, SPIONS as contrast agent	−1 mm (10^−3^–10^−6^) (millimolar level)	No limit	Minutes to hours	High spatial resolution and deep magnetic field penetration tumor tissue specific	Costly low sensitivity long imaging time needs contrast media to enhance signals	Cross sectional images suitable to perform soft tissue imaging (yes)	81, 82
Nuclear medicine imaging	PET (high energy and γ‐rays)	Cu (64), F (18) and Ga (68), SPIONS in combination with Ga (68) and Cu (64) used as radioactive tracer	−4 mm (picomolar level)	No limit	Minutes to hours	Highly sensitivity Fast imaging 3D imaging of body tissue	Expensive, risk of ionizing radiation	This imaging may help to reveal the metabolic and biochemical function of the tissues and organ at molecular and physiological level, (yes)	83, 84, 85
	SPECT (γ ‐rays)	In (111), I (123, 131), Tc (99), used radioactive tracer	−10 mm (picomolar level)	Limitless	Minutes to hours	Whole body with spatial and temporal resolution	Low spatial resolution, expensive, risk of ionizing radiation	This techniques shows how the blood flows to tissue and organ at physiological level (yes)	86, 87
Ultrasound imaging	Ultrasound (high frequency sound)	Microbubble (gas filled) and non‐microbubble type agents	‐	‐	‐	No ionization radiation, good spatial resolution, high specificity and sensitivity. Low cost, safe and fast	Limited penetration/sensitivity (have lower intrinsic specificity for malignant tissues and cannot be used for entire body imaging), operator dependent	Comparatively safer, cheaper and widely used high‐frequency sound waves to view inside the body (yes)	138
Optical imaging	FL imaging (visible or near‐infrared light)	Molecules light sensitive and fluorophores (fluorescence dye e.g. ICG, gold, Cy 5.5, Cy7, Ce6, quantum dots) used as contrast agent	0.3 μm (picomolar level with)	<10 cm	Few seconds to minutes	High sensitivity and spatial resolution Simple and cost‐effective	Limited tissue depth penetration (<5 mm), toxicity and biocompatibility concerns, especially with heavy‐metals quantum dots	Fluorescence provides visualization of biological processes taking place in a living organism (in development)	88, 89, 90, 91, 92
‐	Raman (monochromatic light)	Carbon‐based nanomaterials (carbon dot, carbon nanotubes and graphene oxide)	‐	‐	‐	High specificity, compatible with physiological measurements	Prolonged data acquisition times, sophisticated data analysis	This technique provides images with both spectral and spatial information (in development)	139, 140, 151
Non‐optical imaging	Photoacoustic (PA) imaging	Light absorbates (fluorophore, quencher)	‐	‐	‐	Non‐invasive deep‐tissue imaging with high temporal and spatial resolution	Limited penetration (<5 mm)	Ultrasonic waves are produced by irradiating the material with a pulsed laser. Images are reconstructed energy absorptions and distribution occurs in the tissue at the molecular and physiological levels (in development)	63, 93, 94

Abbreviations: γ, gamma rays; Ce6, Chlorin e6; CMT, Computed tomography; Cy5.5, Cyanine5.5; Cy7, Cyanine7; FL, Fluorescence imaging; Gd, Gadolinium; I, Iodine; ICG, Indocyanine green; In, Indium; Mn, Manganese, MRI, Magnetic Resonance imaging; PA, Photoacoustic imaging; PET, Positron emission tomography; SPECT, Single‐photon emission computed tomography; SPIONS, Superparamagnetic nanoparticle; Tc, Technetium.

Although MR imaging demonstrates high spatial resolution into deeper structures, it is, however, not preferred in all the settings due to poor sensitivity to probes and high cost.^81^, ^82^ On the other hand, CMT gives high resolution images, nonetheless, it is invasive, topped up by its low sensitivity to probes, and poor soft tissue delineation.^78^, ^79^, ^80^ PET and SPECT scans have high sensitivity to probes but show poor resolution and are highly expensive and invasive.^83^, ^84^, ^85^, ^86^, ^87^ Fluorescence and bioluminescence are non‐invasive, have high sensitivity to probes and provide multichannel imaging but do not provide high resolution and deeper level penetration. Two‐photon excited fluorescence can lead to cell destruction with deeper tissue penetration.^88^, ^89^, ^90^, ^91^, ^92^ PA imaging, a non‐invasive technique with high tissue penetration, and high resolution, however, has limited path length due to temperature sensitivity and weak absorption at shorter wavelengths.^63^, ^93^, ^94^ Nanodrug delivery systems can help in overcoming limitations related to imaging and provide more competent and high‐quality imaging. The multicomponent theranostic systems can be efficiently combined within a specific nanoformulation system so that the conjugated nanoparticle can offer companionable theranostic modes. For instance, chemotherapy (chemotherapeutic drugs‐loaded nanoparticles) can be combined with hyperthermia therapy to attain synergistic cancer therapy. Numerous strategies have been reported including combinatorial benefits of therapeutic agents with diagnostic tools to achieve significant results against the carcinogenic cells. The corresponding and coactive performances of these combinatorial strategies significantly enhance the outcomes and lay footing for more effective theranostic platforms.

This review represents and focuses on a recently designed and developed new class of theranostic nanoformulations of triple combination cancer therapy guided by single and/or multi‐model imaging in a single nanocarrier to simultaneously diagnosis and treat cancer (Figure 1 illustrates components of theranostic nanoformulations and their encapsulation methods and Table 2 summarizes triple combination therapeutics with imaging and targeting [optionally] moiety, payload and their stimuli methods for efficient cancer treatment in pre‐clinical studies). The combination of triple therapies (e.g., chemotherapy with photothermal and PDT, radiotherapy with phototherapy and PDT and immunotherapy with phototherapy and PDT or immunotherapy with chemotherapy and phototherapy) would combat the tumor recurrence and show anti‐metastasis effects at very low doses with tumor targeting. In addition, imaging techniques not only provide the real‐time observation of tumor site but also visualize drug distribution and measure therapeutic efficacy. In general, theranostic nanomedicine would improve the anti‐tumor therapeutic efficacy through a synergistic effect.

FIGURE 1(A, B) Pictorial illustration of the theranostic nanoformulation of triple therapeutics for combination cancer therapy: (A) Representation of synergistic multi‐modal‐theranostic nanoformulation and components in a single nanocarrier and application and mode of delivery (intravenous) of synergistic multi‐modal‐theranostic nanoformulation for cancer treatment. (B) Pictorial representation of delivery of three therapeutics with mono and/or multi‐model imaging (optionally targeting) agents in a single nanocarrier: (I) Physical encapsulation: (a) all three therapeutics and imaging agent physically encapsulated simultaneously, (b) all the three therapeutics encapsulated with the help of non‐covalent (electrostatic) interaction, (c) three therapeutics and imaging agent encapsulated by emulsion method, (d) therapeutics and imaging agents physically encapsulated one after other, (e) therapeutics and imaging agents physically encapsulated and targeting moiety by non‐covalent interaction, (f) therapeutics and imaging agents physically encapsulated and targeting moiety attached by chemical conjugation, (g) therapeutics and imaging agents encapsulated by non‐covalent interaction one after other followed by adsorption of the targeting agent on the surface of the nanocarrier. (II) Chemical conjugation: (a) firstly, one therapeutics conjugated to the nanocarrier material then other therapeutics/imaging physically encapsulated, (b) one therapeutics conjugated to the nanocarrier material followed by other therapeutic then imaging agents conjugated. Figures produced using chemdraw software.

**Figure:**
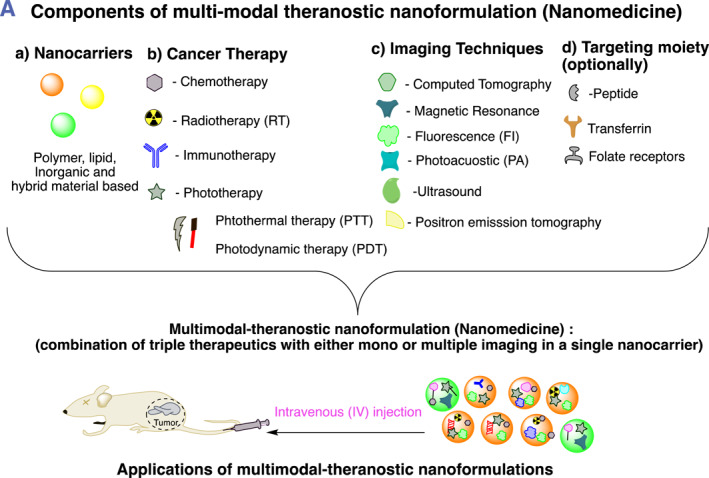

**Figure:**
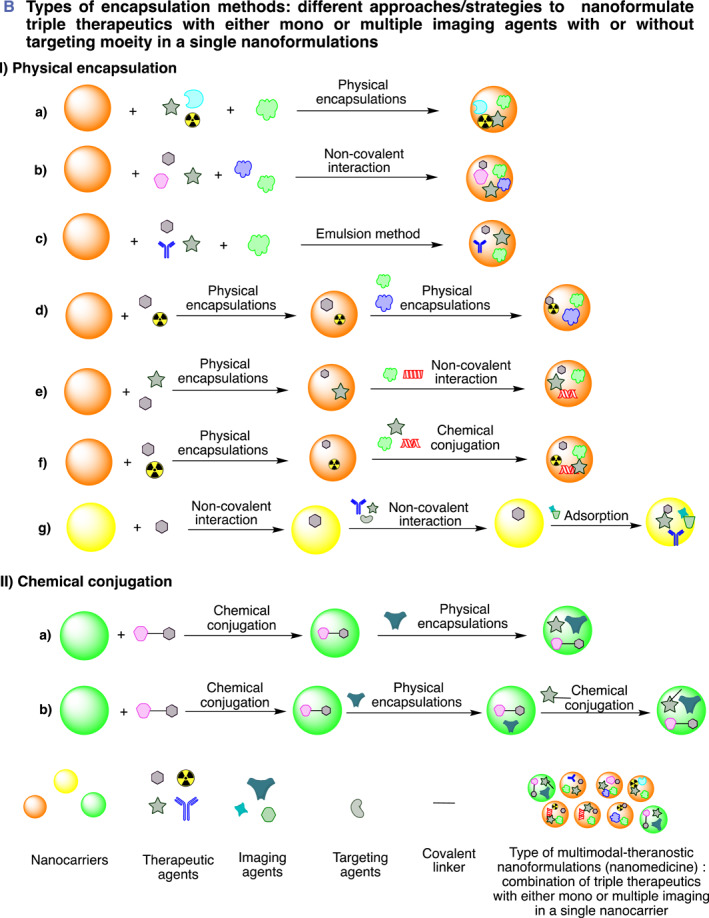

**TABLE 2:** A Summary of recently developed theranostic‐nanoformulations of triple therapeutics guided with single or multiple imaging and optionally targeting groups in a single nanoformulation to diagnose and treat cancer.

	Theranostic nanoformulation (combination of triple therapeutics with single and/or multiple imaging)			Theranostic nanoformulation approach			Payload method	Stimuli	Cancer type (dose)	Therapeutic efficacy	Ref
		Nanocarrier material		Drug/agents	Imaging agents	Target agents					
Pre‐clini cal	Chemotherapy in combination with photothermal therapy (PTT) and photodynamic therapy (PDT)‐imaging modality [mono (FL) or (MR), bimodal (FL/PA), (FL/MR) and triple (FL/PA/MRI) or FL/TP/R] imaging										
	Biomimetic‐hollow‐metal organic nanoparticle (CT/PDT/PTT‐FL)	Zeolitic imidazolate framework‐8 (ZIF‐8)	Dox		ICG	Breast cancer cell	Physical encapsulation	Light	Breast (4T1) cancer (0.4 mg/kg)	Excellent synergism with anticancer and anti‐metastasis effects at a very low dose	71
			ICG								
	Hybrid nanoparticle (CT/PDT/PTT‐MR)	CuCl_2_, Cu_2_O, Fe_2_O_3_, PEG_2000_‐COOH	Dox		Superparamagnetic iron oxide	‐	Physical encapsulation	Light and magnetic	Breast cancer (13 mg/kg)	Remarkable anti‐tumor therapeutic efficacy with synergetic combination therapy	95
			CuS								
	Theranostic nanoprobe (CT/PDT/PTT‐FL)	Gold	Dox		PPa	MMP2 substrate (CGDEVDHGK)	Chemical conjugation and physical encapsulation	pH and light	Liver cancer	Synergistic treatment of triple therapeutics yielded best therapeutics effects with enhanced stability and remarkably reduced non‐specific toxicity of the parent therapeutics.	96
			Pheophorbide a (PPa)								
	Lipid‐inorganic nanoparticles (CT/PDT/PTT‐FL/PA/MR)	Gd, MSNs Cholesterol, DPPC DSPE‐PEG_2000_	Dox		Gd2O3, ICG	Folic acid	Physical encapsulation	Light	Breast (4T1) cancer	Superior tumor targeting and controlled drug release	97
			ICG								
	Inorganic‐carbon dots bioglass (CT/PDT/PTT‐FL/TP/R)	Mesoporous bioglass (calcium and silica) DPBF	Dox		fBGN	‐	Non‐covalent (electrostatic) interaction	pH and light	Cervical carcinoma (5,10 and 20 mg/kg)	Real‐time drug distribution observation, light induced tumor cells destruction and significant improvement in cancer treatment quality.	98
			fBGN								
	Nanoparticles (CT/PDT/PTT‐FL/PA)	Ti {OCH(CH₃) ₂} ₄.	DOX		TiO2 Cy5.5	‐	Physical encapsulation	pH and light	Breast (MDA‐MB‐231) cancer (1 mg/mL)	Superiority of triple therapeutics, tumor growth reduction, and thorough ablation of solid tumor	69
		Dopamine	TiO2‐x				Chemical conjugation				
	Nanocapsule (CT/PDT/PTT‐MR/FL)	PLGA‐ PEG‐NH_2_ PNIPM	Dox		Fe/FeO, ICG	Folic acid	Water/oil/water emulsion	Light and radio wave	Epithelial (KB) carcinoma cells) 20 mg/kg	Achieved imaging‐guided synergetic therapy, enhanced tumor accumulation and improved therapy efficacy	99
			ICG								
			Fe/FeO								
	Radiotherapy in combination with photothermal therapy (PTT) and photodynamic therapy (PDT)‐dual and triple imaging modalities (CT/PA/FI and CMT/PA)										
	Core‐shell inorganic nanohybrid (−30 nm) (RT/PTT/PDT‐PA/CMT/FL)	Tungsten disulfide (WS_2_)	WS2, PANI, Ce6		WS_2_, PANI, Ce6	HA	Physical encapsulation and non‐covalent (electrostatic) interaction	Light	Breast (4T1) cancer (20 mg/kg	Superior tumor growth suppression with reduced systemic toxicity	67
	Inorganic nanoparticle (RT/PTT/PDT‐CMT/PA)	BiOI, Bi_2_S_3_ BSA		‐BiOI, ‐Bi_2_S_3_, ‐BiOI‐	BiOI and BiOI@Bi_2_S_3_		Physical encapsulation	X‐rays and light	Hepatocellular carcinoma (2 mg/mL)	Higher level id ROS generation intracellularly that leads to DNA damage as well as other component in cancer cells slowdown the tumor growth	68
	Immunotherapy in combination with photothermal therapy (PTT) and photodynamic therapy (PDT)‐mono imaging modality (FL) or immunotherapy in combination with chemotherapy and photothermal therapy (PTT)‐Dual imaging modalities (MR/PA and FI/PA)										
	Hybrid‐polymer‐lipid nanocomplex (IT/PTT/PDT‐FL)	Graphene oxide, DSPE, PEG DSPE‐PEG_2000_		Dye‐IR820 CPG ODN	Dye‐IR820	TPP (mitochondria)	Non‐covalent (hydrophobic) interaction and adsorption strategy	Light	Breast (EMT6) cancer (−100 μg/mice)	Remarkable tumor growth inhibition (the tumor inhibition rate −88%) and negligible toxic effects	100
	Biomimetics polymeric core‐shell nanoparticle (IT/CT/PTT‐MR/PA)	PLGA		Imiquimod	Prussian blue	Cancer (4T1) cells membrane	Water/oil/water double emulsion	Light	Breast (4T1) caner) (−200 μL of 3 mg/mL)	Greater inhibition of primary and metastatic tumors	101
				DTX							
				Prussian blue							
	Polymeric nanoparticle (IT/CT/PTT‐FL/PA)	HA		CPT	IRDye‐800 CW	HA	Chemical conjugation, physical encapsulation, and chemical conjugation	Light and enzyme	Breast (4T1) cancer (−100 and 500 μg/mL) ‐	Achieved high‐precision tumor therapy and prevented tumor metastasis and recurrence	102
				Polypyrrole							
				A‐PD‐L1							

Abbreviations: A‐PD‐L1, anti‐Programmed death‐ligand 1; Bi_2_S_3_, Bismuth sulphide; BiOI, Bismuth oxyiodide; BSA, Bovine serum albumin; Ce6, Chlorin e6; CMT, computed tomography; CPG ODN, CpG oligodeoxynucleotides; CT, chemotherapy; Cu_2_O, Copper(I) oxide; CuCl_2_, Copper(II) chloride; Cy5.5, Cyanine5.5; Dox, doxorubicin; DPPC, Dipalmitoyl phosphatidylcholine; DSPE‐PEG_2000_, 1,2‐distearoyl‐sn‐glycero‐3‐phosphoethanolamine–N‐[methoxy(polyethylene glycerol)‐2000]; DTX, Docetaxel; Dye‐IR820, New Indocyanine Green; fBGN, carbon dot mesoporous bioglass nanoparticles; Fe/FeO, Iron/Iron(II) oxide or ferrous oxide; Fe_2_O_3_, Iron(III) oxide or ferric oxide; FL, Fluorescence imaging; Gd, Gadolinium; Gd_2_O_3_, Gadolinium(III) oxide; HA, Hyaluronic acid; ICG, Indocyanine green; IRDye‐800, Phosphoramidite; IT, immunotherapy; MMP 2, matrix metalloproteinase‐2 also known as 72 kDa type IV collagenase and gelatinase A is an enzyme; MRI, Magnetic Resonance imaging; MSNs, Mesoporous silica nanoparticles; PA, Photoacoustic imaging; PANI, Polyaniline; PDT, photodynamic therapy; PEG2000‐COOH, N‐[carboxy(polyethylene glycol)‐2000; PLGA, Poly(lactic‐co‐glycolic acid); PLGA‐PEG‐NH_2_, Poly(L‐lactide‐co‐glycolide)‐block‐poly(ethylene glycol)‐Amine, PEG‐NH_2_‐Poly(ethylene glycol) bis(amine), PNIPAM Poly(N‐isopropylacrylamide); PPa, Pheophorbide a; PTT, phototherapy; R, Raman imaging; RT, radiotherapy; Ti {OCH(CH _3_)_2_}_4_, Titanium(IV) isopropoxide; TiO_2_, titanium dioxide; TP, Two‐photon excitation microscopy (TPEF or 2PEF); WS_2_, Tungsten disulfide; ZIF‐8, Zeolitic imidazolate framework ‐8.

## 2 MECHANISM OF TUMOR TARGETING BY NANOFORMULATION

**FIGURE 2:**
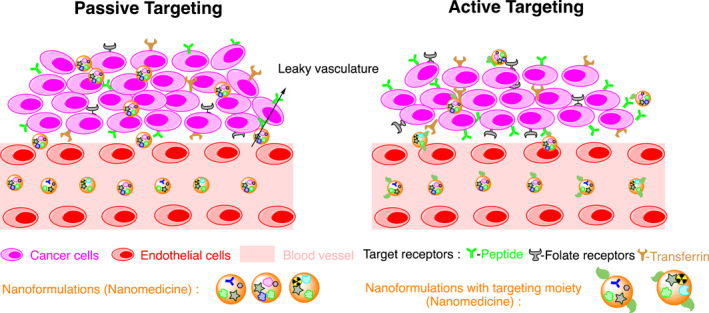
Mechanism for nanodrug delivery system mediated tumor targeting: Passive targeting occurs through the leaky vasculature surrounding the tumor tissues and active targeting occurs through the cross‐interaction between the ligand/molecule on the surface of NFs and the overexpressed targeting receptors of the cell, that is, tumor cells. Figure produced using chemdraw software.

There are various nanoformulations and each uses a different targeting mechanism for drug delivery to tumor sites. Broadly, the nano‐drug vehicles act at the tumor site by two mechanisms, namely active and passive targeting (Figure 2). In passive targeting, NFs are accumulated by exploiting the tumor site characteristics, majorly enhanced permeability and retention (EPR) effect in the TME. The highly porous vasculature of TME supports passive tumor targeting, which is based on the principle of diffusion improving drug efficacy and reducing systemic toxicity. However, the preferential accumulation of nanoformulations is reduced due to high interstitial fluid pressure and abnormality of TME. Hence, different nanocarriers may exploit the other properties of TME such as low pH, varying degree of enzymatic secretions, and higher redox potential for effective tumor targeting. On the other hand, the active targeting is mostly dependent on interplay between the ligand/molecule on the surface of NFs and the overexpressed targeting rectors of the cell, that is, tumor cells. In short, passive targeting is like “missile” and active targeting is more specific and selective.^103^, ^104^, ^105^, ^106^, ^107^, ^108^, ^109^, ^110^, ^111^, ^112^

## 3 THERANOSTIC NANOFORMULATIONS FOR TRIPLE COMBINATION CANCER THERAPY

Numerous multimodal theranostic nanoformulations (presented in Table 2) have been designed and investigated and used to deliver a triple combination of therapies guided by imaging studies (which may vary from single to multiple imaging) in a single nanocarrier for the cancer treatment. These theranostic nanoformulations have been extensively developed based on several materials alone or in amalgamation, such as inorganic materials [for example, copper, iron, gadolinium, tungsten disulfide (WS_2_), iron oxides, mesoporous silica nanoparticles (MSNs), gold, titanium, bismuth], organic materials polymers (hyaluronic acid (HA), PLGA and PEG) and lipids.^67^, ^68^, ^69^, ^71^, ^95^, ^96^, ^97^, ^98^, ^99^, ^100^, ^101^, ^102^ They act in a synergistic manner to increase their therapeutics efficacy. Various theranostic nanoformulations of triple therapeutics are mentioned below in the subsections 3.1, 3.2, 3.3.

### 3.1 Theranostic nanoformulations of chemotherapy, photothermal and photodynamic therapy for cancer therapy

This section describes the design and preparation of nanoformulation (namely biomimetic nanoparticle, hybrid nanoparticle and nanocapsule) of triple therapeutics (chemotherapy with other two therapies e.g., photothermal and photodynamic) and simultaneously mono imaging (MR, FI) and a combination of imaging modalities (FI/MR, FI/PA, MRI/PA/FI, FI/Two photon/Raman imaging) with high‐resolution multimodal imaging of tumor cells in the mice models (pre‐clinically) for the effective cancer therapy. These smart theranostic nanoformulation helps in drug distribution, measuring of therapeutic efficacy and can provide structural, physiological, and molecular information of tumor. Hence, this section highlights the emergence and impact of theranostic nanoformulation of triple therapeutics that showed potential combination cancer therapy.

**FIGURE 3:**
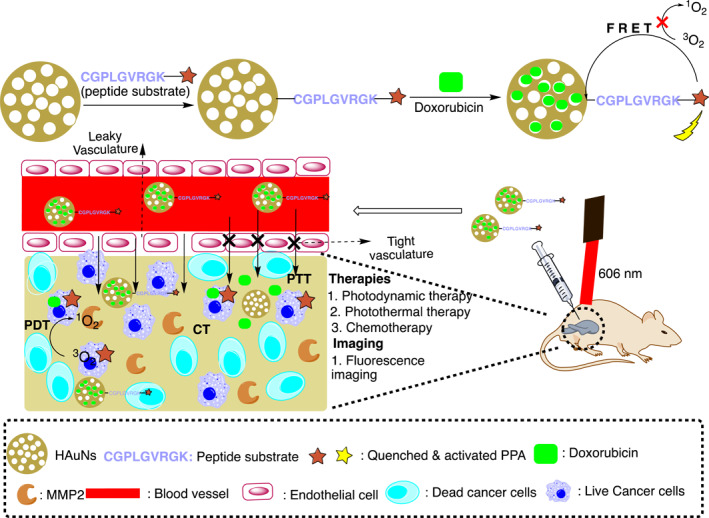
Schematic representation of hollow gold nanosphere for local drug delivery system for fluorescence imaging with triple combination therapies (CT/PTT/PDT) with single light triggered strategy. Reproduced with permission.^96^ Copyright 2019, American Chemical Society. Figure re‐produced using the chemdraw software. CT‐chemotherapy, FRET‐fluorescence resonance energy transfer (FRET), MMP2‐ matrix metalloproteinase‐2, nm‐nanometer, PDT‐Photodynamic therapy, PTT‐photothermal therapy.

A multifunctional nanoplatform of hollow porphyrinic (H‐PMOF) with an inorganic mesoporous spherical shell was developed through a facile self‐sacrificial ZIF‐8 template. It had low dosage DOX and ICG (having drug loading capacity of 635%). Results demonstrated high tumor cell targeting and slower tumor growth in vivo.^71^ In another study, hollow mesoporous copper sulfide nanoparticles (HMCuS NPs) along with iron oxide nanoparticles (IONPs) containing Dox irradiated by NIR demonstrated enhanced effect of phototherapy and excellent antitumor therapeutic efficacy due to coupled plasmonic resonances.^95^ A theranostic nanoprobe containing hollow gold nanospheres (HAuNs), a triple combination of chemo, photothermal and PDT guided by activatable fluorescence for the diagnosis and treatment of cancer, demonstrated low nonspecific toxicity of photosensitizer and enhanced stability of the anticancer drug (Figure 3).^96^

**FIGURE 4:**
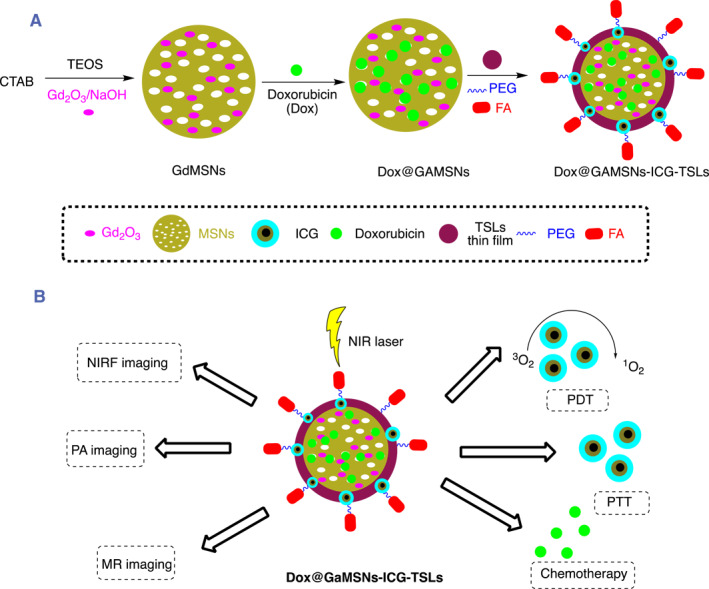
Pictorial and schematic representation of Dox@GdMSNs‐ICG‐TSLs nanoformulation of triple combination therapies (CT/PDT/PTT) with single light triggered guided by triple (FL/PA/MR) imaging: (A) preparation, (B) functions of Dox@GdMSNs‐ICG‐TSLs nanoformulation. Reproduced with permission.^97^ Copyright 2017, American Chemical Society. Figure re‐produced using the chemdraw software. CTAB, cetyltrimethylammonium bromide; FA, folic acid; Gd_2_O_3_, gadolinium(III) oxide; ICG, indocyanine green; MSNs, mesoporous silica nanoparticles; NaOH, sodium hydroxide; NIR, near infrared; NIRF, near infrared fluorescence imaging and MR‐magnetic resonance imaging; PA, photoacoustic imaging; PDT‐Photodynamic therapy; PEG, poly(ethylene glycol); PTT, photothermal therapy; TEOS, tetraethyl orthosilicate.

A theranostic nanocomposite based on Gd doped‐MSNs, DOX and ICG loaded thermosensitive liposome (DOX@GdMSNs‐ICG‐TSLs), guided by tri‐modal imaging under NIR irradiation (Figure 4), appeared to be promising as a bridge for cancer treatment.^97^

**FIGURE 5:**
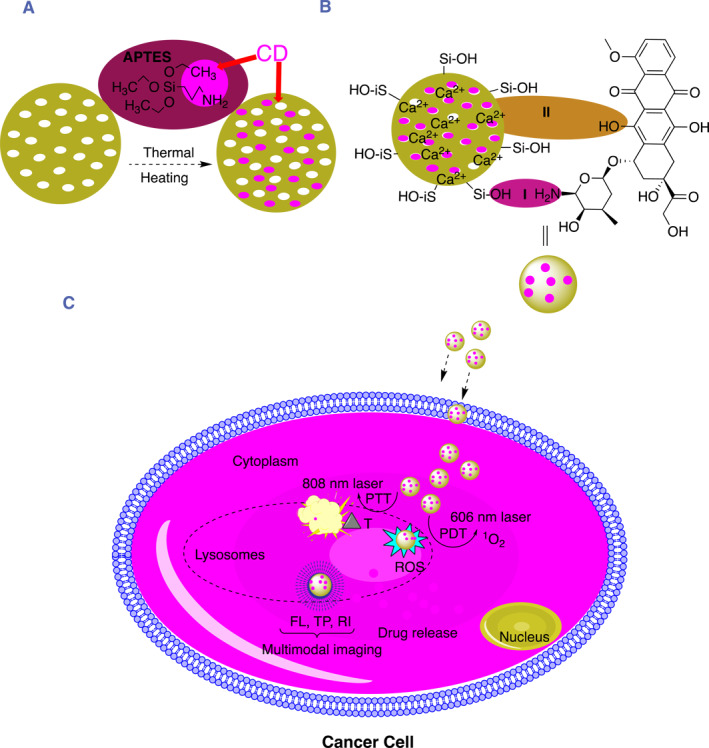
Pictorial and schematic representation of hollow gold nanosphere for local drug delivery system for fluorescence imaging with triple combination therapies (PDT/CT/PTT) with single light triggered. (A) Development of DC‐based bio glass (B) Mechanism of doxorubicin high loading and slow release and (C) drug delivery, tri‐modal imaging and phototherapy. Reproduced with permission.^98^ Copyright 2020, American Chemical Society. Figure re‐produced using the chemdraw software. APTES, (3‐Aminopropyl)triethoxysilane; CD, carbon dots; FL, fluorescence imaging; PDT, photodynamic therapy; PTT, photothermal therapy; RI, Raman imaging; ROS, reactive oxygen species; TP, two photon imaging.

Rajendra kumar singh et al, attempted to develop a multicolor fluorescent bioglass nanoparticle (fBGn) with FL, two‐photon (TP), and Raman imaging (RI), which demonstrated photothermal and photodynamic effects on cancer cells (Figure 5). The fBGn was effective in delivering pH‐dependent DOX and hence, this multifunctional fBGn was found potentially useful for cancer theranostic.^98^

**FIGURE 6:**
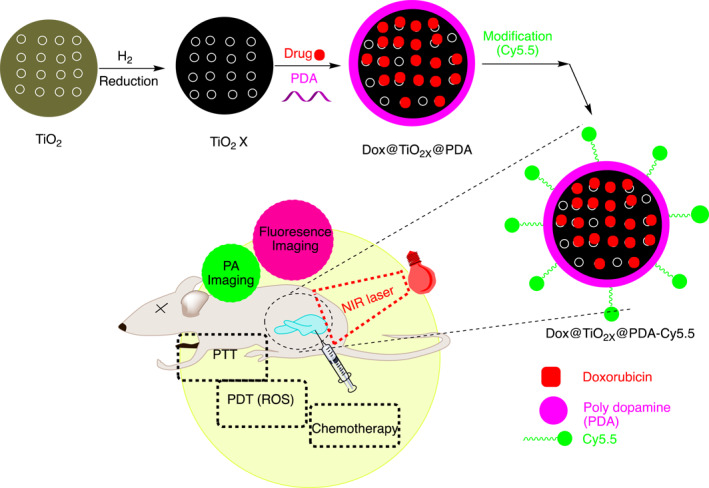
Pictorial and schematic representation of hollow gold nanosphere for local drug delivery system for fluorescence imaging with triple combination therapies (CT/PTT/PDT) with single light triggered. Reproduced with permission.^69^ Copyright 2017, American Chemical Society. Figure re‐produced using the chemdraw software. Cy5.5, cyanine5.5; Dox, doxorubicin; NIR, near infrared; PA, photoacoustic imaging; PDT, photodynamic therapy; PTT, photothermal therapy; ROS, reactive oxygen species; TiO2, titanium dioxide.

Wei Guo et al., prepared TiO_2‐x_ based theranostic system guided by bimodal imaging and NIR‐triggered triple‐therapy of CT/PTT/PDT, which showed the superiority of triple therapy in the inhibition and ablation of solid tumors, and B‐mode ultrasonography confirmed the same findings through a liquefaction necrosis process (Figure 6).^69^

Wang Z et al. designed and developed a smart nanocapsule (DOX‐ICG@Fe/FeO‐PPP) guided by dual‐mode MRI and FI under NIR irradiation. The hallmark of this nanocapsule was effective circulation and high stability in the blood due to its large size in the beginning; NIR irradiation reduced and decomposed the size of tumor and help in controlled release of DOX. Fe/FeO helped in the overproduction of ROS for correcting hypoxia of tumor to manage the hypoxia‐related resistance during chemo/photo and chemodynamic therapy. This NP subsequently proved to be highly effective as a therapeutic and diagnostic strategy and has a high potential for clinical translation of smart nanocapsule.^99^

### 3.2 Theranostic nanoformulations of radiotherapy with photothermal and photodynamic therapies for cancer therapy

This section briefly summarizes the relevant nanotheranostic studies related to the triple combination of radiotherapy, photothermal and PDT.

Wang J et al., engineered a multifunctional nanohybrid based on chlorin e6 (Ce6), polyaniline (PANI), HA and WS_2_ nanodot (HA‐WS2@PANI/Ce6) encompassing radio/photothermal/photodynamic (RT/PTT/PDT) therapies via multi‐modality guided imaging [fluorescence (FL), photoacoustic (PA), and computed tomography (CMT)] for cancer treatment. This nanohybrid had the core features of good dispersibility, NIR‐region absorbance and large X‐ray attenuation coefficient of tungsten, better ROS productivity and excellent biocompatibility, embarking its potential in combination therapy in the future. The findings revealed in vivo enhanced tumor uptake, improved tumor retention and diagnostic effect after intravenous injection, proving it to be a potential strategy for tumor treatment.^67^ Another theranostic nanoformulation, namely semiconductor heterojunction nanoparticles (SHNPs)‐BiOI@Bi_2_S_3_@BSA (bovine serum albumin) with a multifunctional theranostic nanoplatform for synergistic RT/PTT/PDT therapies via dual bioimaging of CMT/PA was designed. This radiosensitizer‐based nanocomposite had fewer side effects and better outcome.^68^

### 3.3 Theranostic nanoformulations of immunotherapy in combination with chemotherapy and photothermal therapy for cancer treatment

This section briefly summarizes the relevant nanotheranostic studies and recent advances related to triple combination of IT, chemotherapy, and phototherapy for combination cancer therapy.

**FIGURE 7:**
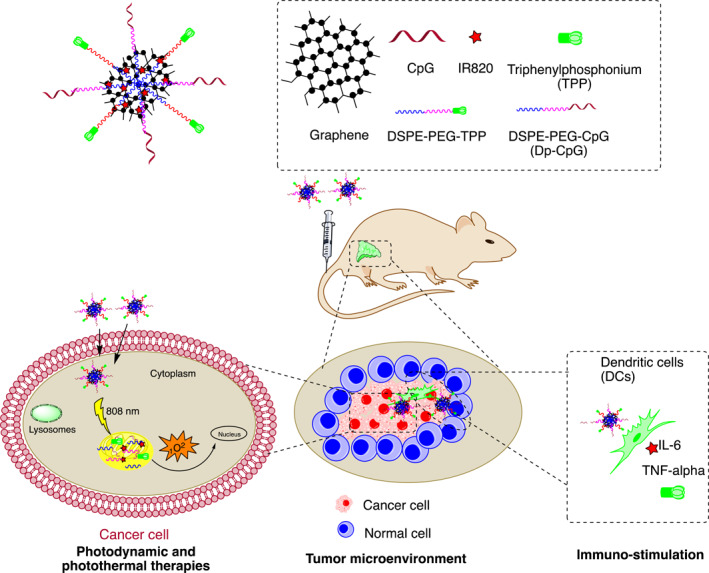
Schematic diagram of the theranostic nanoformulations for cancer therapy. Reproduced under terms of the CC‐BY license.^100^ Copyright 2021, The Authors, published by Springer Nature. Figure re‐produced using the chemdraw software. DSPE‐PEG, 1, 2‐distearoyl‐sn‐glycero‐3‐phosphoethanolamine‐poly(ethylene glycol); dye‐IR820, new indocyanine reen; TNF alpha, tumor necrosis factor alpha.

Chunhui et al. designed mitochondria targeted and NIR light‐activatable multifunctional nanographene including triple punch of PDT, PTT, and immunotherapy. In addition, a new immunostimulatory conjugate DP‐CpG was introduced. As a result, DP‐CpG enhanced tumor immunogenicity due to the secretion of proinflammatory cytokines (e.g., IL‐6, TNF, and IFN). The NIR laser and the photoactive nanocomplex demonstrated abundant ROS and tumor suppression rate of 88% with no adverse effects on mice (Figure 7).^100^

**FIGURE 8:**
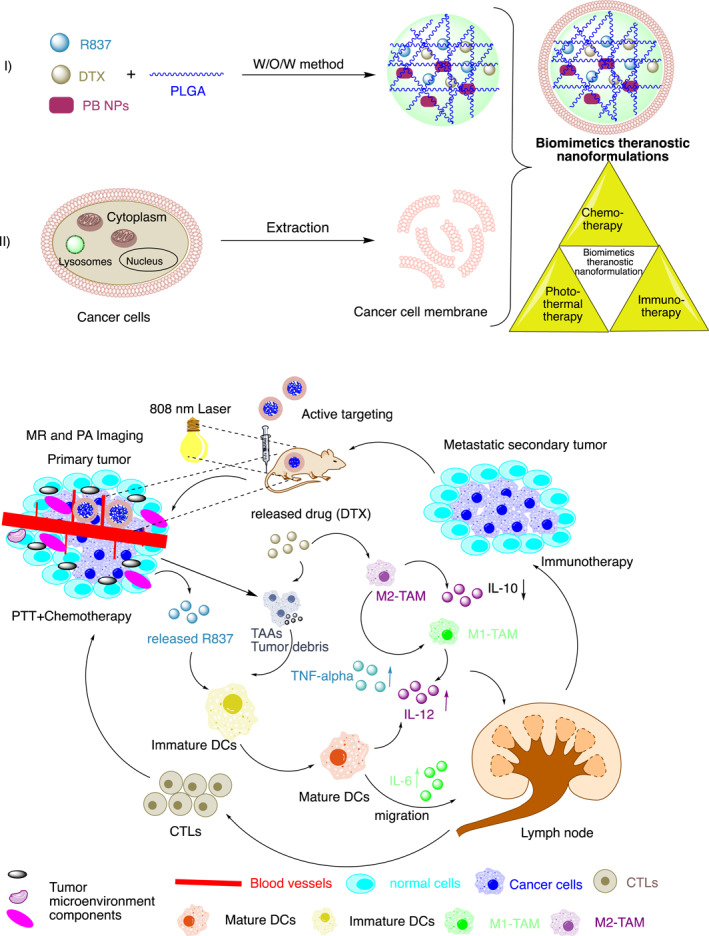
Pictorial and schematic representation of hollow gold nanosphere for local drug delivery system for fluorescence imaging with triple combination therapies (IT/CT/PTT) with single light triggered. Reproduced with permission.^101^ Copyright 2018, American Chemical Society. Figure re‐produced using the chemdraw software. CTL‐tumor antigen‐specific cytotoxic T lymphocyte; DTX, docetaxel; IL‐10, interleukin 10; IL‐6, interleukin 6; MR, magnetic resonance imaging; PA, photoacoustic imaging; PB NPs, prussian blue nanoparticles; PLGA, poly(lactic‐co‐glycolic acid); PTT, photothermal therapy; R837, imiquimod; TAM, tumor‐associated macrophages; TNF, alpha‐tumor necrosis factor alpha; w/o/w, water in oil‐in‐water emulsions methods.

A “Nano‐targeted cells” with (4.18 mg DTX) and imiquimod‐immune adjuvant (R837, 1.57 mg) loaded in Prussian blue 2.98 mg, photothermal transduction agents (PTAs)‐ in the core guided by bimodal PA‐MR imaging demonstrated the combined effect of DTX and PTT in terms of in situ tumor eradication and immunotherapy enhanced the maturation rate of dendritic Cells by 4.34‐times compared to the control (Figure 8). Furthermore, the infiltration of cytotoxic T lymphocytes improved/increased from 17.33% (control) to 35.5% leading to significant inhibition of primary tumor and metastasis.^101^

**FIGURE 9:**
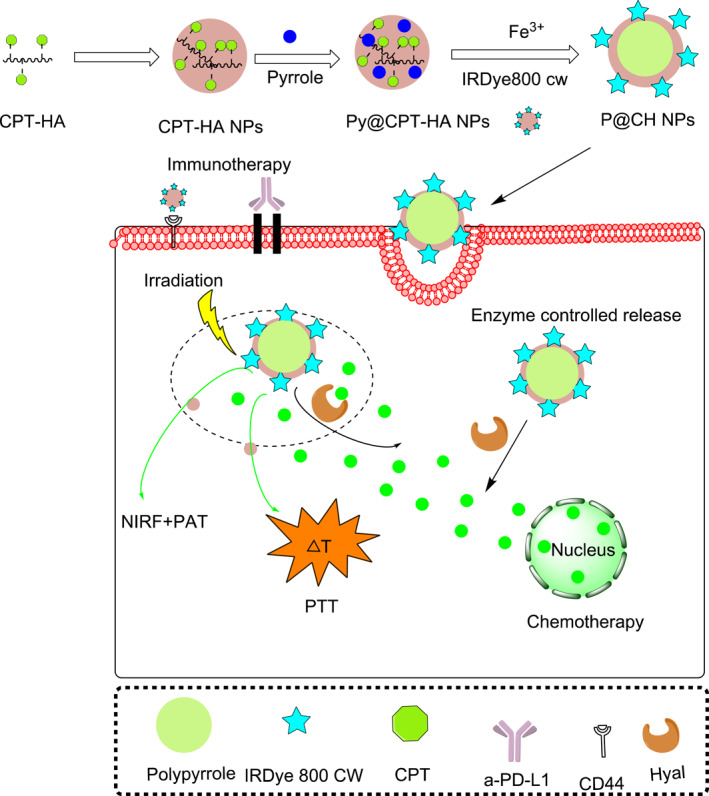
Schematic illustration of triple combination therapies, including synergetic chemo‐PTT in combination with immunotherapy guided by near‐infrared FI and PA imaging. Reproduced under terms of the CC‐BY license.^102^ Copyright 2019, The Authors, published by Elsevier. Figure reproduced using the Chemdraw software. A‐PD‐L1, anti‐L1‐antiprogrammed death‐ligand 1; CD44, cell surface adhesion receptor; CPT, camptothecin; dye‐IR820, new indocyanine green; FL, fluorescence imaging; HA, hyaluronic acid; Hyal, hyaluronidase enzyme; PTT, photothermal therapy.

Sun Wei et al. 2019 developed a novel polypyrrole (PP) nanoparticle (PPy@CPT‐HA‐IRDye800CW) by conjugating a camptothecin (CPT)‐HA shell using the near infrared dye IRDye800CW. Triple therapy of PTT, CT and IT was administered guided by FI and PA imaging. As a result, breast cancer was completely removed through an enhanced anti‐tumor immune response with no metastasis or recurrence in mice bearing 4T1 tumor (Figure 9).^102^

#### 3.3.1 Summary of theranostic nanoformulations from Section 3 and its subsections

Cancer nano‐theranostic has been considered a promising and potential approach to provide reliable diagnosis and management. The field of clinical diagnosis has been extremely important in fighting against cancer as well as in assessing tumor progression and regression. The hallmark of the aforementioned NFs was the selection of appropriate nanomaterials and imaging techniques with their unique characteristics.^113^, ^114^, ^115^, ^116^, ^117^, ^118^ The ideal multi‐modality theranostic nanoformulations would be the those having enhanced tumor uptake, maximum biocompatibility, strong near infrared (NIR) absorbance, good dispersibility, greater ROS productivity, absent or minimal premature drug leakage, negligible multidrug resistance, absence of recurrence, zero probability of metastasis, remarkable cancer cell death, enhanced therapeutic efficacy and better prognosis. For instance, WS_2_ nanodot uses high X‐ray absorption coefficient of tungsten, HA helps in active tumor targeting, and PANI shell overcomes the limitations of WS_2_ nanodot for radio‐photodynamic‐photothermal (RT/PDT/PTT) therapies guided by FL/PA/CMT.^67^ Multimodality imaging is a better and comprehensive tool for theranostic. For instance, NIRF imaging has high sensitivity and PA is excellent in spatial resolution. Polypyrrole has higher biocompatibility and high efficiency for energy conversion and can be used to enhance tumor targeting effect as a photothermal agent.^102^ NIR light promotes CPT release in tumor by its high PTT effect. In addition, the strong affinity and high specificity of HA and CD44 are considered to design a novel theranostic PPy@CPT‐HA‐combined with immunotherapy in the above cases. Immune check point blockers, when combined with PTT or chemotherapy, inhibit the recurrence and metastasis by inhibiting T cell activation and thus activating the immune response. This might prove to be an important alternative for the clinical management of solid tumor such as breast cancer. Research in the field of interaction between nanomaterials and x‐rays is in its infancy, and effective radiosensitizers are still a challenge, especially one integrating with multiple treatment modalities. For instance, a photocatalytic semiconductor, Bismuth oxyiodide (BiOI), has been exploited as a radiosensitizer as it contains many high Z‐elements, which can enhance the radiotherapeutic effect.^68^ In addition, BiOI act as an excited PS and produce highly reactive species to kill cancer cells as evident from above cases.^68^ The role of superparamagnetic IONPs has been exemplary as a gatekeeper modification in hollow mesoporous copper sulphide nanoparticles (HMCuS NP_S_) to prevent the premature drug leakage application. Controlled on‐demand drug release and higher photothermal effects have been demonstrated in a short time with minimal side effects in NIR‐responsive HMCuS/DOX@IONP‐PEG for chemo‐phototherapy guided by MRI.^95^ Gadolinium (Gd) doped MSN nanocomposite showed promising findings as an attractive nano system along with exploitation of indocyanine Green (ICG) which can be used in near infrared fluorescence (NIRF) and PA and generate high ROS as well. ICG has a significant role in the PDT and PTT effects, NIRF and PA.^97^ Carbon‐dots (CD) can be exploited for multimodal imaging agents owing to their lower toxicity, light resistance, ease of functioning, tunable absorption emission spectra etc.^98^ In addition, silica‐based mesoporous bio glass having a high drug loading capacity, tunable pore size, excellent biocompatibility can be used to develop CD‐based bio glass nanotheranostic carriers for targeted delivery, real‐time monitoring, and phototherapy. Nanoparticles of the hydrogenated black titanium dioxide, which is not extensively explored for cancer treatment, were recently used for dual cancer imaging by FL/PA and NIR triggered CT/PDT/PTT.^69^ Similarly, TiO2‐x sphere matrix can be used for extensive optical absorbance in the NIR region, producing hyperthermia and ROS after NIR irradiation.^69^ Overall, these theranostic‐nanoformulations having a robust feature of drug incorporation, imaging movement, unique photosensitizer, PTT, and PDT activity help in implementing successful multi‐modal theranostic in cancer treatment with an improved therapeutic index. These theranostic‐nanoformulations may help in better prognosis for the cancer patients, ultimately improve the biodistribution (in vivo) and have an accurate approach to visualize and monitor the therapeutics.

## 4 CLINICAL STATUS AND CHALLENGES OF NANOFORMULATION FOR CANCER TREATMENT

In the last decades, a variety of potential nanoformulations have emerged and found the most effective and safer therapeutics, which enhance therapeutic efficacy and reduce the toxicity.^56^, ^119^, ^120^, ^121^, ^122^, ^123^, ^124^, ^125^, ^126^ These NFs function as a smart, intelligent, and efficient nano‐vehicle, containing active ingredients/drugs (single or combination of therapeutics) which travel throughout the human body (stay and available for longer time within the human body) and reach the target areas in respective diseases. However, poorly designed non‐smart NFs lead and promote delivery to healthy cells and cause adverse drug reactions. In addition, considering the physiochemical properties (e.g., size, chemistry, and surface charge), these NFs would be modified to control the blood circulation and tissue penetration. For instance, positive charge surface NFs reveal greater uptake than negative charge NFs due to the electrostatic interaction between the cell membrane and NFs. Our previous review described the combined cancer therapy (chemo and antiangiogenic therapies) in a single nanocarrier. These nanocarriers were made up of several organic, inorganic, or hybrid materials and the active drugs were entrapped with help of physical adsorption, encapsulation, and chemical conjugation strategies to co‐deliver the two therapeutics in controlled drug release manner at target sites.^127^ These NFs demonstrated tumor growth inhibition, low drug resistance and overall improved treatment efficacy in mice models.

**FIGURE 10:**
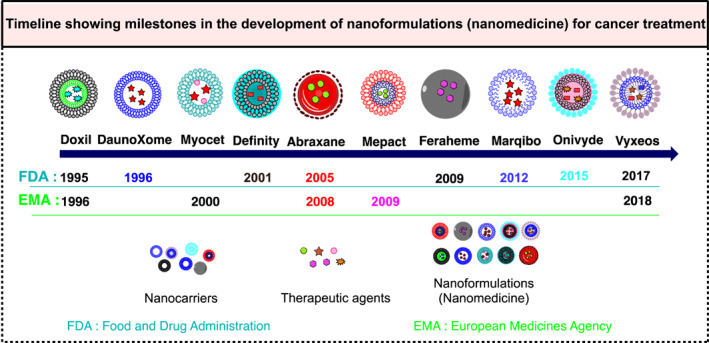
Timeline showing milestones in the design and development of nanoformulation (nanomedicine) for cancer diagnosis and treatment. Figure produced using chemdraw software.

There are several nanoformulations which have been designed and investigated in the last 50 years (Figure 10 explaining the timeline and Table 3 represent the status of NFs for cancer treatment). Some of these NFs are approved and currently available in the market and others are not approved by regulatory agencies e.g., Food and drug administration (FDA), European Medicines Agency or respective countries.^128^, ^134^, ^135^, ^136^, ^137^

**TABLE 3:** Current status of the nanoformulation (nanomedicine) for cancer therapy.

Nanoformulation name	Nanoformulation type	Therapeutics used	Indication	Status	Ref
Doxil^®^	Liposome	Doxorubicin	Kaposi's sarcoma	Approved by FDA in 1995 and EMA in 1996	128, 129
DaunoXome^®^	Liposome	Daunorubicin	Leukemia	Approved by FDA in 1996	128, 130
Myocet^®^	Liposome		Various cancer: Breast, lymphoma or ovarian	Approved by EMA in 2000	128, 131
Definity^®^	Lipid microsphere	Perflutren	Ultrasound enhancement for liver or breast, liver cirrhosis	Approved by FDA in 2001	128
Abraxane^®^	Albumin‐particle nanoparticle	Paclitaxel	Various cancers including solid malignancies breast, lymphomas, bladder, lung, pancreatic melanoma, or liver	Approved by FDA in 2005 and EMA in 2008	128
Mepact^®^	Liposomal formulation	Mifamurite	Osteosarcoma	Approved by EMA in 2009	128
Feraheme^®^	Iron polyglucose colloid formulation	Sorbitol carboxymethyl ether	Iron deficient anemia imaging: Brain metastases lymph node metastases, neuroinflammation in epilepsy	Approved by FDA in 2009	128
Marqibo^®^	Liposomal formulation	Vincristine	Lymphoma, brain, leukemia, or melanoma	Approved by FDA in 2012	128
Onivyde^®^	Liposomal formulation	Irinotecan	Solid malignancies, pancreatic, sarcomas, or brain	Approved by FDA in 2015	128
Vyxeos^Tm^	Liposomal formulation	Daunorubicin + cytarabine	Acute myeloid leukemia	Approved by FDA in 2017 and EMA in 2018	128, 132, 133

Abbreviations: EMA, The European Medicines Agency; FDA, The United States Food and Drug Administration.

Despite the various advantages and the fact that nanoformulation (nanomedicine) is one of the most advanced methodologies in the creation of novel cancer therapies, very few nanocarriers‐based cancer medicines have been successfully tested in humans and reached the market, for instance, Doxil® was the first FDA‐approved nano‐drug in year 1995. It has the advantages of prolonged drug circulation time and effective tumor targeting. It has been successfully used in various kinds of cancers, for instance, Kaposi sarcoma. However, its patent expired in 2010, and there has been no FDA‐approved generic “Doxil” available since then. Other nanoformulations such as Abraxane® and DaunoXome® have shown improved therapeutic efficacy with low toxicity of the parent drug.^88^, ^138^ Abraxane, also known as nab‐paclitaxel (protein bound paclitaxel), has been used against pancreatic cancer, metastatic breast cancer, and non‐small cell lung cancer as a first‐line treatment. It has proven to be more effective than conventional paclitaxel‐containing metastatic breast cancer patients and furthermore, patients need not undergo pre‐medication to prevent any hypersensitivity reactions. For instance, Abraxane combined with gemcitabine has shown better patient survival than gemcitabine alone in patients with metastatic adenocarcinoma of the pancreas. Overall, on a risk‐benefit analysis, the benefits of Abraxane are greater than its risks.

Similarly, polymer‐paclitaxel conjugate is in Phase III trial and has low toxicity compared to free and parent paclitaxel.^89^, ^90^ Similarly, FDA approved CPX‐351, namely Vyxeos® (with cytarabine and daunorubicin ratio of 5:1), is used for the treatment of acute myeloid leukemia.^91^, ^139^ Likewise, a liposome‐based nanoformulation with irinotecan and floxuridine is also reported to be in phase III for the treatment of advanced colorectal cancer.^140^

In today's time, the challenges exist in the form of designing a smart nanoformulation for successful clinical translation. This can be brought on by thorough assessment of the safety and toxicity profile of these smart nanoformulations. In addition, safety measures such as extensive pre‐clinical evaluation, pharmacokinetics and pharmacodynamics, rigorous evaluation of smart nanoformulations for systemic toxicity, immunotoxicity, targeted toxicity, long‐term safety, and compliance to regulatory bodies can build up a strong body of evidence. This would create a robust and dedicated system for this technological innovation.

The success rate of conventional monotherapy and combined therapy is very limited due to systemic toxicity. Additionally, these therapies have failed to maintain and deliver optimized drug dosages and have negligible efficacy. For instance, gemcitabine is used in metastatic breast cancer, however the median survival time is not prolonged in these patients. With nanotechnology advancement, polymeric nanoparticles of HA containing synergistic pairs of gemcitabine and imiquimod enhanced the anticancer activity.^141^

## 5 CONCLUSIONS AND FUTURE OUTLOOK

This review has summarized the recent progress and impact in the field of synergistic triple combination cancer therapy guided by mono, dual and multi‐modal imaging techniques in a single nanocarrier for precise cancer diagnosis and therapy. Various smart materials used for nanocarriers, such as WS_2_ nanodot, HA, PANI shell, porphyrinic mesoporous shell, gold nanospheres, PEG etc. have been effectively used owing to their individual unique characteristics and compatibility with remarkable therapeutics. Cancer remains one of the most formidable challenges in modern medicine, necessitating innovative and multifaceted strategies for improved treatment outcomes. Traditional cancer therapies for cancer, such as chemo, radio, and surgery, often entail considerable side effects and limited specificity, underscoring the need for precision medicine approaches. In response to this imperative, the concept of theranostic has emerged as a transformative paradigm, integrating diagnostics and therapeutics within a single platform to enable personalized and targeted cancer management.

Theranostic nanoformulations represent a cutting‐edge facet of this approach, offering a versatile toolkit to address the complexities of cancer treatment. These nanoformulations encapsulate therapeutic agents, such as chemotherapeutics or targeted therapies (PDT/PTT), alongside diagnostic components, typically imaging agents, or biomarkers. By combining diagnostics and therapeutics, theranostic nanoformulations hold the promise of optimizing cancer therapy in unprecedented ways. While the multimodality theranostic approach of cancer management based on novel nanoformulations presents a promising approach to cancer therapy and imaging, it's essential to acknowledge its limitations. In vivo biocompatibility, toxicity, target specificity, excretion after therapeutic/imaging function, clinical translation, cost, accessibility, and interdisciplinary collaborations are some of the important challenges to be addressed before successful clinical implementation of these multimodality treatment strategies using nanocarriers. Future research should focus on mitigating these limitations, optimizing the strategy, and conducting rigorous clinical trials to assess its safety and efficacy in real‐world patient populations. In conclusion, above‐mentioned theranostic nanoformulation in the field of synergistic triple combination cancer therapy guided by mono, dual and multi‐modal imaging techniques in a single nanocarrier serves for precise cancer medicine. Biological evaluations (In in vitro and in vivo) of these multimodal theranostic nanoformulations showed promising results and particularly in preclinical mice model with excellent antitumor efficiency and satisfactory imaging effects. Briefly, in vivo studies showed the improved tumor retention and tumor growth suppression with fewer adverse drug reactions and no tumor recurrence and metastasis. Based on these positive results, further exploration could be tested in other animals depending on the protocol and eventually among cancer patients for efficient cancer treatment.^142^, ^143^ Although these multimodal theranostic nanoformulations exhibit specific characteristics of drug assimilation and shield, imaging activity, unique photosensitizer, photothermal and photodynamic activity with improved therapeutic efficacy and safety profile. However, cost‐effectiveness needs to be weighed while designing and producing it for clinical use in the future for these theranostic nanoformulations. Nevertheless, there is still a long way to go to the market. Collectively, these multimodal‐theranostic nanoformulations would serve as an ideal and potential nanoplatform for precise cancer treatment and hopefully would have clinical success in terms of patient outcome.^144^, ^145^ Moreover, the clinical success of cancer nanomedicine is very challenging, and the goal is to deliver adequate therapeutic to tumor and its associated microenvironment. Further studies are warranted for artificial intelligence (AI) driven physiologically based pharmacokinetics (PBPK) model to predict nanoformulation delivery to tumor in mice. It could serve as a platform for early screening with an aim to reduce the number of animal use. Furthermore, 3D cell culture models could be explored for these nanoformulations.^146^, ^147^, ^148^, ^149^, ^150^

## AUTHOR CONTRIBUTIONS

Hongbo Zhang and Amit Kumar Rajora conceived the idea; Amit Kumar Rajora, Eknath Damu Ahire, Manju Rajora and Sukhvir Singh wrote the paper. Amit Kumar Rajora, Manju Rajora, Jaydeep Bhattacharya and Hongbo Zhang reviewed and edited the paper. All authors have read and agreed to the current version of the manuscript.

## CONFLICT OF INTEREST STATEMENT

Hongbo Zhang is an executive editor for *Smart Medicine* and was not involved in the editorial review or the decision to publish this article. All authors declare that there are no competing interests.
